# Expression of peptidyl-prolyl isomerase PIN1 and its role in the pathogenesis of extrahepatic cholangiocarcinoma

**DOI:** 10.3892/ol.2013.1525

**Published:** 2013-08-12

**Authors:** URANGOO JAMIYANDORJ, JUN SANG BAE, SANG JAE NOH, SARANGEREL JACHIN, JI EUN CHOI, KYU YUN JANG, MYOUNG JA CHUNG, MYOUNG JAE KANG, DONG GEUN LEE, WOO SUNG MOON

**Affiliations:** Department of Pathology, Chonbuk National University, Medical School, Research Institute of Clinical Medicine and Research Institute for Endocrine Sciences, Jeonju, Chonbuk 561-756, South Korea

**Keywords:** extrahepatic cholangiocarcinoma, peptidyl-prolyl isomerase PIN1, phosphorylation

## Abstract

The phosphorylation of proteins on serine/threonine residues that immediately precede proline (pSer/Thr-Pro) is a key signaling mechanism by which cell cycle regulation and cell differentiation and proliferation occur. The peptidyl-prolyl isomerase PIN1-catalyzed conformational changes of the pSer/Thr-Pro motifs may have profound effects on the function of numerous oncogenic and cell signaling pathways. To date, no studies have examined the expression of PIN1 and its potential role in the pathogenesis of extrahepatic cholangiocarcinoma (ECC). Therefore, the present study performed an immunohistochemistry analysis of the expression of PIN1 in 67 cases of ECC and evaluated its association with clinicopathological factors. In addition, the role of PIN1 was examined using synthetic small interfering RNA (siRNA) to silence PIN1 gene expression in human CC RBE cells. Positive PIN1 expression was observed in 35 of the 67 (52.2%) ECC cases and was predominantly localized to the nucleus of the tumor cells. The immunoreactive score for PIN1 was significantly higher in the tumor cells (4.07±0.4) compared with the adjacent benign bile duct cells (1.19±0.4) (P<0.001). PIN1 expression was significantly correlated with tumor cell proliferation (Ki-67 labeling index; P=0.024). Silencing PIN1 expression using siRNA significantly decreased the proliferation, migration and invasion of the tumor cells. In conclusion, the results indicated that the expression of PIN1 may play a key role in the development and progression of ECC.

## Introduction

Cholangiocarcinoma (CC) is the primary epithelial cell cancer of the biliary tract and the second most prevalent form of primary hepatic tumor (1). Due to a late diagnosis and no effective therapy, CC is associated with a high rate of mortality and a poor prognosis (1). Little is known about the molecular changes and mechanisms that are involved in the pathogenesis and pathophysiology of CC.

Phosphorylation on serine or threonine residues that precede proline (pSer/Thr-Pro) represents a key regulatory mechanism for controlling the function of signaling molecules in a cellular process. The pSer/Thr-Pro exists as two types of conformations that are regulated by the peptidyl-prolyl isomerase, PIN1. PIN1 changes its conformation and thereby regulates the function of phosphoproteins (2–4). PIN1-mediated prolyl-isomerization potentiates the progression of the cell cycle and cell proliferation through the regulation of target genes, including cyclin D, cyclin E, jun, myc and TP53 (3,4). Whether PIN1 acts as a tumor promoter or suppressor remains a controversial issue. PIN1 may play a positive or negative role in tumorigenesis, possibly in a cell-type selective manner (4). To date, however, no studies have examined the significance of the expression and the role of PIN1 in the pathogenesis of extrahepatic CC (ECC).

The present study examined PIN1 expression in surgical human ECC specimens and its association with clinicopathological factors. Furthermore, the associations between PIN1 expression and the rate of cellular proliferation (Ki-67 index) and between PIN1 expression and the expression of TP53, a tumor suppressor gene, were also examined. Finally, the effects of silencing PIN1 using small interfering RNA (siRNA) on the growth, migration and invasion of CC cells was investigated.

## Materials and methods

### Patients and specimens

The present study was approved by the Institutional Review Board (IRB) of Chonbuk National University Hospital (Jeonju, Chonbuk, South Korea). Written informed consent was waived by the IRB due to the retrospective nature of the study. A total of 67 ECC specimens and their corresponding non-neoplastic tissues that were obtained from patients who underwent surgical resection at the Chonbuk National University Hospital between 1998 and 2009 were used to examine the location and expression of PIN1. For immunohistochemical staining, 10% formalin-fixed, paraffin-embedded tissue sections were prepared as 4-μm thick tissue samples. Of the 67 patients with ECC, 49 (73.1%) were male and 18 (26.9%) were female. The tumors were histologically graded as 24 cases of well-differentiated (35.8%), 38 cases of moderately-differentiated (56.7%) and five cases of poorly-differentiated (7.5%) ECC. The clinicopathological data were obtained from the patients who were previously hospitalized at Chonbuk National University Hospital using a retrospective review of the medical records. The tumors were staged according to the 2010 American Joint Committee on Cancer tumor-node-metastasis classification system (5).

### Cell lines and culture

The human CC RBE cell line was purchased from the RIKEN BRC cell bank (Tsukuba, Japan). The cell line was maintained in RPMI-1640 medium (Gibco, Grand Island, NY, USA) supplemented with 10% fetal bovine serum (FBS) and penicillin/streptomycin (Gibco), cultured at 37°C and 5% CO_2_ in a humidified incubator.

### Immunohistochemistry

The immunohistochemical staining was performed by a polymer intense detection system using the Bond-Max Automatic Stainer (Leica, Bannockburn, IL, USA), according to the manufacturer’s instructions. Briefly, following antigen retrieval (microwaved at high power for 10 min in 0.01 M citrate buffer; pH 6), the samples were incubated with anti-PIN1 (Santa Cruz Biotechnology, Santa Cruz, CA, USA), anti-Ki-67 (Novocastra, Newcastle, UK) and anti-TP53 (Novocastra) antibodies for 30 min. Peroxidase activity was detected using the enzyme substrate, 3-amino-9-ethyl carbazole. The samples were subjected to an immunohistochemistry analysis and their immunohistochemical properties were rated according to a score that was calculated by multiplying the intensity of the stain with the area of the stain. The intensity of the cell staining was graded as follows: 0, no staining; 1+, weak staining; 2+, moderate staining; and 3+, strong staining. The area of staining was evaluated as follows: 0, 0–9% of cells stained positive; 1+, 10–29% of cells stained positive; 2+, 30–69% of cells stained positive; and 3+, >70% of cells stained positive. The maximum combined score was nine and the minimum score was zero. If the combined score was ≥4, the tumor was considered positive. Otherwise, the tumor was considered negative. The samples with nuclear Ki-67 and TP53 staining of >20% of the tumor cells were defined as positive.

### siRNA transfection

siRNA was used to silence PIN1 expression. PIN1 siRNA and negative controls were purchased from Bioneer Corporation (Daejeon, Korea). The sequences for PIN1 specific siRNA were forward, 5′-CCAUUUGAAGACGCCUCGU(dTdT)-3′ and reverse, 5′-ACGAGGCGUCUUCAAAUGG(dTdT)-3′. The sequences for the negative control were forward, 5′-CCUACGCCACCA AUUUCGU(dTdT)-3′ and reverse, 5′-ACGAAAUUGGUGGCGUAGG(dTdT)-3′.

Transfection of the siRNA was performed using the lipofectamine RNAiMAX transfection reagent (Invitrogen, Carlsbad, CA, USA), following the manufacturer’s instructions.

### Western blotting

Western blotting of PIN1 in the RBE cell line was performed as previously described (6). Briefly, the cell lysates were subjected to denaturing sodium dodecyl sulfate-polyacrylamide gel electrophoresis (SDS-PAGE) followed by electroblotting and immunoblotting for anti-PIN1 (Santa Cruz Biotechnology). The blots were developed using horseradish peroxidase conjugated anti-mouse IgG secondary antibodies (Santa Cruz Biotechnology) and the immune complexes were visualized using an enhanced chemiluminescence detection system (Amersham Biosciences, Buckinghamshire, UK). The blots were then analyzed using an LAS-3000 luminescent image analyzer (Fuji Film, Tokyo, Japan).

### Cell growth and proliferation assay

Cell growth was determined using the colorimetric tetrazolium-derived 3-(4,5-dimethylthiazol-2-yl)-2,5-diphenyltetrazoniumbromide (MTT) assay (Sigma, St. Louis, MO, USA). DNA synthesis of the cells was assessed by the bromodeoxyuridine (BrdU) incorporation assay (Roche Applied Science, Mannheim, Germany). For the cell growth and proliferation assay, 48 h after the transfection of the siRNA, the cells of each group were reseeded in 96-well plates at a density of 5×10^3^ cells/well. Following 24–120 h, the MTT and incorporated BrdU were measured colorimetrically using a Bio-Rad model 680 microtiter plate reader (Bio-Rad, Hercules, CA, USA) at wavelengths of 560 and 450 nm, respectively.

### In vitro assay of cellular migration and invasion

Cellular migration and invasion were assayed using a 24-transwell migration chamber (Corning Life Sciences, Acton, MA, USA) and a 24-transwell BioCoat Matrigel invasion chamber (BD Biosciences, San Jose, CA, USA) with an 8-μm pore size polyvinylpyrrolidone-free polycarbonate membrane, respectively, following the manufacturers’ instructions. The cells that migrated to or invaded the lower surface of the filter were counted under a light microscope at ×100 magnification in five randomly selected fields per well.

### Statistical analysis

The statistical analysis was performed using SPSS version 15.0 (SPSS Inc., Chicago, IL, USA). Data are presented as mean ± standard deviation. The clinicopathological characteristics were compared with PIN1 expression using the χ^2^ test. P<0.05 was considered to indicate a statistically significant difference.

## Results

### PIN1 expression in the ECC tissue samples

**Figure 1:**
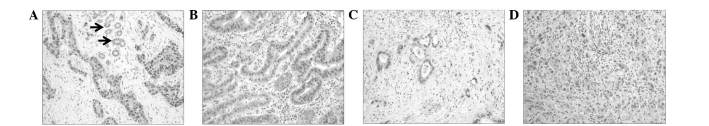
Immunohistochemical expression of PIN1 in extrahepatic cholangiocarcinoma (ECC). Tumor cells showing a strong immunoreactivity for PIN1 in the nuclei of the tumor cells, while the benign bile duct cells are negative for PIN1 expression (arrows). (A,B) Well- (C) moderately- and (D) poorly-differentiated ECCs. Magnification, ×200.

Of the 67 ECC tissue sections, 35 were PIN1-positive (52.2%). The immunoreactive score for PIN1 was significantly higher in the tumor cells (4.07±0.4) compared with that of the adjacent non-neoplastic bile duct cells (1.19±0.4; P<0.001). In the CC cells, PIN1 expression was predominantly localized to the nucleus. However, in certain cells, it was present in the nucleus and the cytoplasm (Fig. 1).

### Correlation between PIN1 expression and clinicopathological factors

PIN1 expression in ECC and its correlation with the clinicopathological factors are presented in Table I. PIN1 expression was significantly correlated with the cell proliferative rate (Ki-67 labeling index; P=0.024). However, PIN1 expression was not significantly correlated with other clinicopathological parameters, including tumor differentiation, lymph node metastasis, distant metastasis, nerve invasion, gross type and tumor stage. The positive expression of TP53 was observed in 52.2% (35/67) of the total cases of ECC. There was no significant correlation between PIN1 and TP53 expression in the ECC cases.

### Effect of PIN1 silencing on cell growth, proliferation, migration and invasion

**Figure 2:**
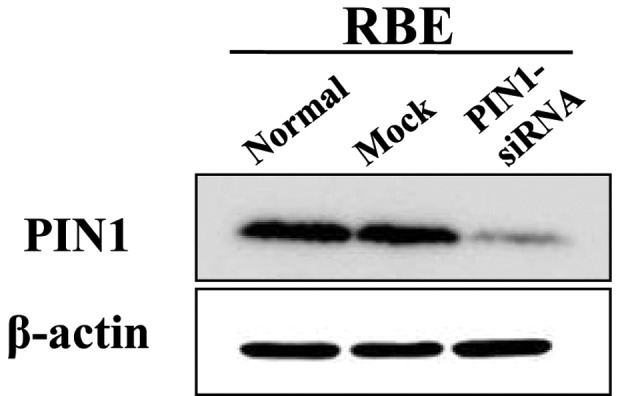
Western blot analysis of PIN1 in the RBE cell line. The RBE cells that were transfected with PIN1 siRNA showed a decreased expression of PIN1 protein. siRNA, short interfering RNA.

**Figure 3:**
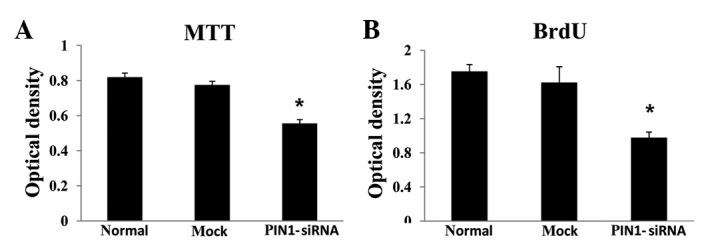
MTT and BrdU proliferation assay in the RBE cell line. (A) MTT assay showing the PIN1-downregulated siRNA-transfected RBE cells with a significantly decreased cell growth compared with the control (P<0.05). (B) BrdU assay showing the PIN1-downregulated cells with a significantly decreased rate of cell proliferation compared with the control (P<0.05). The experiment was performed in triplicate. MTT, 3-(4,5-dimethylthiazol-2-yl)-2,5-diphenyltetrazonium bromide; BrdU, bromodeoxyuridine; siRNA, short interfering RNA.

**Figure 4:**
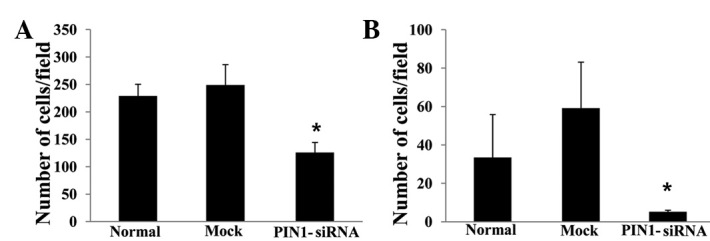
*In vitro* assay of cell migration and invasion. (A) Cell migration of the PIN1-silenced RBE cells was decreased by 1.9-fold compared with the control (P=0.006). (B) The invasion ability of the PIN1-silenced RBE cells was decreased by 11.3-fold compared with the control (P<0.005). The experiment was performed in triplicate. siRNA, short interfering RNA.

The transfection with PIN1 siRNA resulted in a marked decrease in the expression of PIN1 at 48 h post-transfection in the RBE cells (Fig. 2). Silencing PIN1 gene expression in the RBE cells using PIN1 siRNA resulted in a significant inhibition of cell growth compared with the control (P<0.05; Fig. 3A). In the PIN1 siRNA-transfected cells, there was a significant decrease in the BrdU incorporation compared with the control (P<0.05; Fig. 3B). Silencing PIN1 gene expression significantly inhibited the migration (Fig. 4A) and invasion abilities of the RBE cells (Fig. 4B).

## Discussion

pSer/Thr-Pro is a key signaling mechanism by which cellular proliferation and transformation occur. PIN1 is a peptidyl-prolyl isomerase that recognizes the pSer/Thr-Pro motifs in certain proteins and catalyzes the prolyl isomerization (2–4). The prolyl-isomerization induces conformational changes and has a distinct effect on various target genes, including cyclin, β-catenin, jun, myc and p53. These targets of PIN1 play a key role in the regulation of the cell cycle and are often deregulated in cancer (2–4,7–11). The possible involvement of PIN1 in human carcinogenesis is based on the observations that the overexpression of the gene is frequently identified in human cancers (4,7–11). Therefore, PIN1 has been of increasing interest as a potential target in the treatment of patients with cancer. The results of the present study demonstrated that the degree of PIN1 expression was significantly elevated in the ECC tissues compared with the non-tumorous bile duct cells. A significant correlation was identified between the degree of PIN1 expression and the tumor cell proliferation rate (Ki-67 labeling index). PIN1 silencing had a significant inhibitory effect on the growth and proliferation of the ECC cells and reduced their migration and invasion abilities. However, a significant correlation between PIN1 and TP53 expression in the ECC cells was not identified using immunohistochemistry. The results of the present study indicated a possible role for PIN1 in ECC development and also indicated that PIN1 expression may be involved in the proliferation and invasion of tumor cells.

Numerous studies have indicated that PIN1 plays a role in oncogenesis and that the depletion of this gene expression leads to a decreased susceptibility to oncogenesis (7–13). By contrast, several studies have shown that decreased levels of PIN1 are associated with a selective growth disadvantage due to an increase in the time that is required for the progression of the cell cycle (4,14,15). PIN1 is able to promote the degradation of c-Myc and cyclin E with the mediation of the FBXW7 E3 ligase, thus providing the evidence that it acts as a tumor suppressor (16,17). The present data revealed that the degree of PIN1 expression was significantly higher in the ECC tissues compared with the non-tumor tissues. Furthermore, there was a positive correlation between the expression of PIN1 and the Ki-67 labeling index in the ECC cells. The suppression of PIN1 expression decreased the degree of cellular growth and proliferation. The degree of PIN1 expression has been reported to increase in various types of cancer, including human prostate (11,12), breast (10,11) and lung (11,18) cancer and hepatocellular carcinoma (11,13). PIN1 is also involved in increasing tumor cell growth and colony formation through the upregulation of the expression of β-catenin and cyclin D1 in several types of cancer (12,13,18). In addition, it has also been suggested that PIN1 expression may play a role as a prognostic indicator in human cancers occurring in the breast (10), lung (18) and prostate (19). These studies and the results of the present study support the argument that PIN1 overexpression may play a role in promoting the occurrence of tumors and then increase the risk of developing cancer. However, this remains somewhat controversial. By contrast, previous studies have also shown that the downregulation of PIN1 expression is frequently observed in renal cell carcinoma (RCC) and gastric and testicular cancer (11,20). Furthermore, although the expression of PIN1 is frequently observed in patients with Merkel cell carcinoma, those with a higher degree of PIN1 expression have been reported to have a significantly longer overall survival compared with those with a lower degree of expression (21). However, the variability in the biological and clinical effects of PIN1 depending on the types of cancer remains unclear. Teng *et al* demonstrated that PIN1 had an inhibitory effect on the development of RCC where TP53 signaling remained intact. These findings are consistent with the physiological role of PIN1 in regulating the functions of TP53 (20). Several studies have indicated that PIN1 may inhibit the cell cycle and cell growth by stabilizing p53, a tumor suppressor gene (4,14,15,22,23), or by destabilizing oncoproteins (16,17). This indicates a possible correlation between the PIN1 and TP53 expression status in tissue samples of ECC. The p53 gene product is unstable with a short half-life. This explains the reason that wild-type p53 is not well detected. However, a mutation in the p53 gene often results in a prolonged half-life with a loss of function compared with the wild-type allele (25). The mutated p53 gene products tend to accumulate in the cell nuclei and display positive nuclear staining on immunohistochemistry, thus suggesting the presence of mutated p53. The present study demonstrated that TP53 expression was observed in 35 of 67 (52%) surgical ECC specimens using immunohistochemistry. This degree of expression is consistent with an earlier study showing that the mean degree of TP53 expression was 51% (range, 19–86%), as observed by immunohistochemistry in cases of ECC (26). Despite a previous study stating that there was a correlation between PIN1 expression and TP53 in cases of lung cancer (27), the present study failed to identify any significant correlations between the expression of the two genes. It is noteworthy that the overexpression of PIN1 is frequently observed in human cancers where a p53 mutation is prevalent, including breast and liver cancer (10,13,20). Furthermore, PIN1 cooperates with mutant p53 during ras-dependent transformation and increases the aggressiveness through the inhibition of antimetastatic factor p63 and the induction of a mutant p53 transcriptional program in breast cancer cells (24). Based on a previous study demonstrating that TP53 overexpression is frequently observed in cases of ECC (26), we speculate that a p53 mutation inhibits the activity of PIN1 as a tumor suppressor gene and that this may lead to its increased activity in promoting tumor development in ECC cells. However, further studies are warranted to clarify the molecular mechanisms by which PIN1 plays a variable role in the development of diverse types of cancer cells, which would be essential for disclosing its role in association with p53.

The morbidity and mortality of cancer is predominantly based on the invasion and metastasis of cells from the primary occurrence. Since the invasion and metastasis of cancer depends on the migratory and invasive potential, the present study examined whether silencing PIN1 expression in CC cells was able to reduce migration and invasion. The results revealed that the migration and invasion abilities of the cancer cells were significantly reduced with the RNAi-mediated knockdown of PIN1 expression in the RBE cells. This is consistent with a previous study, in which the migration and invasion abilities of lung cancer cells were significantly reduced with the inhibition of PIN1 expression using PIN-1-specific siRNA (18). With the blockage of PIN1 by stable transfection of a microRNA (miRNA) plasmid targeting PIN1, the proliferation and invasion of cells from the malignant melanoma A275 cell line were significantly reduced (28). Similarly, with the depletion of PIN1 by small hairpin RNA, the transforming growth factor (TGF)-β-mediated migration and invasion of human prostate cancer cells (PC3) were significantly inhibited (29). Overall, the present results indicate a potential role for PIN1 expression in the migration and invasion of ECC cells. However, further studies are warranted to examine the mechanisms by which the role of PIN1 in the invasion and metastasis of the tumor is promoted.

In conclusion, the present data indicated that the upregulation of PIN1 is involved in the development and progression of ECC tumors. Silencing PIN1 gene expression significantly inhibited tumor cell proliferation and decreased the migration and invasion of the ECC cells. The high degree of TP53 expression in ECC cells may play a role in promoting the oncogenic potential of PIN1 in these cells.

## Figures and Tables

**Table I:** Expression of PIN1 in ECC and its correlation with the clinicopathological parameters, Ki-67 labeling index and TP53 expression.

Category	Total, n	PIN1 expression, n (%)		P-value

		Negative	Positive	
Differentiation				0.784
Well	24	12 (50.0)	12 (50.0)	
Moderate	38	17 (44.7)	21 (55.3)	
Poor	5	3 (60.0)	2 (40.0)	
T category				0.834
T1, T2	41	20 (48.8)	21 (51.2)	
T3, T4	26	12 (46.2)	14 (53.8)	
LN metastasis				0.13
Absent	51	27 (52.9)	24 (47.1)	
Present	16	5 (31.3)	11 (68.7)	
Distant metastasis				0.569
Absent	62	29 (46.8)	33 (53.2)	
Present	5	3 (60.0)	2 (40.0)	
Nerve invasion				0.582
Absent	27	14 (51.9)	13 (48.1)	
Present	40	18 (45.0)	22 (55.0)	
Gross type				0.285
IG	21	8 (38.1)	13 (61.9)	
PI	46	24 (52.2)	22 (47.8)	
Stage				0.883
I	34	17 (50.0)	17 (50.0)	
II	23	10 (43.5)	13 (56.5)	
III	5	2 (40.0)	3 (60.0)	
IV	5	3 (60.0)	2 (40.0)	
Ki-67 labeling index, %				0.024
<20	15	11 (73.3)	4 (26.7)	
≥20	52	21 (40.4)	31 (59.6)	
TP53 expression				0.183
Negative	32	18 (56.3)	14 (43.7)	
Positive	35	14 (40.0)	21 (60.0)	

ECC, extrahepatic cholangiocarcinoma; LN, lymph node; IG, intraductal growth; PI, periductal infiltrative.
